# Ultra-Processed Food Consumption and Adult Mortality Risk: A Systematic Review and Dose–Response Meta-Analysis of 207,291 Participants

**DOI:** 10.3390/nu14010174

**Published:** 2021-12-30

**Authors:** Wanich Suksatan, Sajjad Moradi, Fatemeh Naeini, Reza Bagheri, Hamed Mohammadi, Sepide Talebi, Sanaz Mehrabani, Mohammad ali Hojjati Kermani, Katsuhiko Suzuki

**Affiliations:** 1 Faculty of Nursing, HRH Princess Chulabhorn College of Medical Science, Chulabhorn Royal Academy, Bangkok 10210, Thailand; wanich.suk@pccms.ac.th; 2 Halal Research Center of IRI, FDA, Tehran 314715311, Iran; 3 Nutritional Sciences Department, School of Nutritional Sciences and Food Technology, Kermanshah University of Medical Sciences, Kermanshah 6718773654, Iran; 4 Department of Clinical Nutrition, School of Nutritional Science, Tehran University of Medical Science, Tehran 1449614535, Iran; Naeini_F34@yahoo.com (F.N.); mohamadihd@gmail.com (H.M.); talebisepide7@gmail.com (S.T.); 5 Department of Exercise Physiology, University of Isfahan, Isfahan 8174673441, Iran; will.fivb@yahoo.com; 6 Department of Clinical Nutrition, School of Nutrition and Food Science, Isfahan University of Medical Sciences, Isfahan 8174673461, Iran; sanaz_mehr6500@yahoo.com; 7 Clinical Tuberculosis and Epidemiology Research Center, National Research Institute of Tuberculosis and Lung Diseases (NRITLD), Masih Daneshvari Hospital, Shahid Beheshti University of Medical Sciences, Tehran 1983535511, Iran; IMHOJJATI@gmail.com; 8 Faculty of Sport Sciences, Waseda University, 2-579-15 Mikajima, Tokorozawa 359-1192, Japan

**Keywords:** ultra-processed food, mortality risk, systematic review, dose–response, meta-analysis

## Abstract

We performed a systematic review and dose–response meta-analysis of observational studies assessing the association between UPF consumption and adult mortality risk. A systematic search was conducted using ISI Web of Science, PubMed/MEDLINE, and Scopus electronic databases from inception to August 2021. Data were extracted from seven cohort studies (totaling 207,291 adults from four countries). Using a random-effects model, hazard ratios (HR) of pooled outcomes were estimated. Our results showed that UPF consumption was related to an enhanced risk of all-cause mortality (HR = 1.21; 95% CI: 1.13, 1.30; I^2^ = 21.9%; *p* < 0.001), cardiovascular diseases (CVDs)-cause mortality (HR = 1.50; 95% CI: 1.37, 1.63; I^2^ = 0.0%; *p* < 0.001), and heart-cause mortality (HR = 1.66; 95% CI: 1.50, 1.85; I^2^ = 0.0%; *p* = 0.022), but not cancer-cause mortality. Furthermore, our findings revealed that each 10% increase in UPF consumption in daily calorie intake was associated with a 15% higher risk of all-cause mortality (OR = 1.15; 95% CI: 1.09, 1.21; I^2^ = 0.0%; *p* < 0.001). The dose–response analysis revealed a positive linear association between UPF consumption and all-cause mortality (*P*_nonlinearity_ = 0.879, *P*_dose–response_ = *p* < 0.001), CVDs-cause mortality (*P*_nonlinearity_ = 0.868, *P*_dose–response_ = *p* < 0.001), and heart-cause mortality (*P*_nonlinearity_ = 0.774, *P*_dose–response_ = *p* < 0.001). It seems that higher consumption of UPF is significantly associated with an enhanced risk of adult mortality. Despite this, further experimental studies are necessary to draw a more definite conclusion.

## 1. Introduction

Food consumption patterns have changed dramatically around the world. Globally, consumption of ultra-processed foods (UPFs) has risen in most middle- or high-income countries and gradually displaced fresh and minimally processed foods [1,2]. The NOVA classification system was first used in 2010 to categorize foods according to their processing level [3,4,5]. The system was last updated in 2016 and classified foods into four categories: unauthorized or minimally processed foods, processed culinary ingredients, processed foods, and UPFs [6]. Many studies have assessed the amount of energy consumed from UPFs in different countries. Accordingly, the consumption of UPFs accounts for approximately 25–60% of total energy intake using individual-level data [7,8,9,10]. UPF energy consumption was highest in the USA and the United Kingdom, while the lowest levels were found in Italy [11]. UPFs comprise a large proportion of almost all ingredients derived from foods and additives, with little or even zero whole-food content. Food substances that are used in UPFs are rare or underutilized for culinary purposes, including added sugar such as fructose and high-fructose corn syrup, oils modified by chemical reactions such as hydrogenated oil, proteins like hydrolyzed protein, casein, whey, and cosmetic additives including thickeners, colors, and emulsifiers [6]. They are typically ready-to-consume foods, have relatively low price, are tasty and energy-dense, and are packaged attractively [6,12].

Globally, the incidence of noncommunicable diseases (NCDs) continues to rise. Over 80% of all premature deaths worldwide are caused by cardiovascular diseases (CVDs), respiratory disease, cancer, and diabetes. Consequently, assessing the impact of various risk factors, including UPF consumption, on mortality from NCDs will be effective in making policies to achieve the World Health Organization (WHO) goal of a relative reduction of 25% in NCDs\ mortality by 2025 [13]. Adding industrialized processed foods to people’s dietary habits has increased the risk of NCDs, the leading cause of mortality. Evidence has shown that a higher intake of UPFs is associated with an increased risk of diabetes, CVDs, cancer, obesity, and other health disorders [4,14,15,16]. The association between UPF intake and risk of mortality was assessed in some studies. The result of a longitudinal study in the USA revealed a positive association between UPF consumption and risk of death due to CVDs and heart disease; however, this association was not ruled out for mortality from cerebrovascular disease [17]. Inconsistent with this result, analysis of NHANCE III could not find any association between UPF intake and CVD mortality, although all-cause mortality was positively associated with UPF intake using a median follow-up period of 19 years [18]. In line with the result of this study, analysis of the Seguimiento Universidad de Navarra (SUN) cohort study revealed no association between UPF consumption and death due to CVDs and cancer disease. However, a dose–response relationship was found between UPF consumption and a higher risk of all-cause mortality [19]. Others have also shown a positive association between higher consumption of UPFs and higher risk of all-cause mortality [20,21,22]. A possible explanation for developing NCDs and mortality risk among people consuming UPFs is their nutritional characteristics. Low micronutrients, vitamin density, and fiber, and high amounts of energy, saturated fat, salt, and added sugar make these foods nutritionally poor [23]. Subsequently, health concerns arise from the high consumption of UPFs. In addition to their low nutritional value, UPFs contain harmful compounds to health, including bisphenols, phthalates, heterocyclic amines, polycyclic aromatic hydrocarbons, furans, and others produced during processing and packaging. These characteristics have been linked to several NCDs, leading causes of death [24,25,26]. 

As a requirement to improve food availability arose in the past decades, the processing of food emerged as a way to do so. Therefore, the effects of UPF consumption on people’s health must be clarified. Therefore, we pooled the findings from observational studies to perform a systematic review and dose–response meta-analysis to determine if UPF intake is associated with mortality risk. It is hoped that the findings of this study will help make the right decisions and policies regarding the use of UPFs and reduce the risk of death from NCDs.

## 2. Materials and Methods

This study was carried out according to the 2020 Preferred Reporting Items for Systematic Reviews and Meta-Analyses (PRISMA) guidelines [27]. The present study protocol was submitted and confirmed in the international prospective register of systematic reviews database (PROSPERO) under registration number CRD42021273097.

### 2.1. Literature Search and Selection

A systematic literature search was performed using ISI Web of Science, PubMed/MEDLINE, and Scopus until 30 August 2021 without language or date limitations. Search terms were a combination of free-text terms and controlled vocabulary related to UPF and mortality, including ((“fast foods”)All Fields) OR “fast foods”)MeSH Terms) OR “ultra processed food*”(All Fields) OR “ultraprocessed food*”(All Fields) OR “ultra processed food*”(All Fields) OR “processed food*”(All Fields) OR “ultra-processed”(All Fields) OR “ultraprocessed”(All Fields) OR “ultra-processed”(All Fields) OR “NOVA”(All Fields) OR “nova food classif*”(All Fields) OR “nova food*”(All Fields) OR “nova food classif*”)All Fields) OR “NOVA food classification system”)All Fields)) AND (“Mortality” (MeSH Terms) OR “Mortality”(Title/Abstract) OR “Death”(Title/Abstract) OR “Fatal”(Title/Abstract) OR “survive”(Title/Abstract) OR “survival”(Title/Abstract)), (Supplementary Table S1). The search strategy for gray literature consisted of a manual search of all original studies cited in the retrieved review studies. 

### 2.2. Inclusion and Exclusion Criteria

The inclusion criteria consisted of the following: observational studies (cohort, case–control, or cross-sectional studies) undertaken in adults (≥18 years) that reported on the association between UPF consumption and the risk of mortality, and that provided effect estimates in the form of hazard ratio (HR), relative risk (RR), or odds ratios (OR) with 95% confidence interval (95% CI). Studies performed in children and adolescents (<18 years), reviews, conference letters, notes, reports, short surveys, and case reports were excluded. The population, intervention, comparator, and outcome (PICO) can be found in Supplementary Table S2.

### 2.3. Study Selection

The evaluation of titles and abstracts and the full-text review process for studies retrieved through our search strategy were conducted individually by two investigators (S.M. and H.M.). Any discrepancies regarding the inclusion and exclusion of selected articles were resolved by consensus or discussion. A standardized process was used according to the inclusion and exclusion criteria, which took into consideration the setting, population, and evaluated exposure(s) and outcome(s) of individual studies.

### 2.4. Data Extraction

A standardized method was also used for the data extraction process undertaken separately by two investigators (S.M. and H.M.). The following information was extracted: (a) the first author’s name; (b) year of publication; (c) country and setting of the study; (d) the number of participants; (e) age; (f) gender; (g) follow-up duration in cohort studies; (h) methods for evaluating exposure; (i) study’s main findings; (j) covariates used for adjustments in the multivariable analyses. Any discrepancies about data extraction were resolved by consensus or discussion with a third investigator (S.T).

### 2.5. Quality Assessment

Two investigators (S.M. and H.M.) separately assessed the quality of each study using the Newcastle–Ottawa Scale (NOS) [28]. The NOS was designed to examine the quality of nonrandomized studies as fit for meta-analyses, and it assigns a maximum of nine points for the least risk of bias in three broad domains: study group selection (four points); study group comparability (two points); exposure and outcome ascertainment for case-control or cohort studies, respectively (three points). Disagreements that were decided by the consensus outcome of the quality assessment for each study are shown in Table 1.

### 2.6. Data Synthesis and Statistical Analyses

We performed statistical analyses with STATA v14.0 (StataCorp, College Station, TX, USA) and SPSS v25.0 (IBM, Armonk, NY, USA). The OR and its 95% CI were assumed as the effect size. The effect estimates reported by the original studies and considered for inclusion in our meta-analyses included OR and HR (and their 95% CI) [29]. The synthesized effect estimates for the current study were expressed as pooled HR with 95% CI. Due to anticipated heterogeneity between studies, the effect estimates were calculated using a weighted random-effects model using the DerSimonian–Laird approach [30]. First, we conducted a pairwise meta-analysis by combining the effect sizes for the highest and lowest categories of UPFs consumption. Heterogeneity among the studies was examined by the Cochran Q and I-squared (I^2^) statistics. The I^2^ value was calculated as ([Q − df])/Q × 100%, where Q is the χ^2^ value and df represents the corresponding degrees of freedom. The heterogeneity was considered significant where the Q statistics were significant (*p* < 0.01) or I^2^ > 50%; more specifically, low, moderate, high, and extreme heterogeneity was defined according to the I^2^ statistic cutoffs of <25%, 25–50%, 50–75%, and >75%, respectively. In addition, we conducted subgroup analyses to evaluate any possible effects of participants’ body mass (less than 25 or more than 25), UPF assessment tools (24 h food records or food frequency questionnaires), follow-up duration (less than 10 years or more than 10 years), and region (America or Europe) on the association between exposures and outcomes.

Sensitivity analysis was carried out by removing each study and recalculating the pooled effect estimates (i.e., one study removed analysis). Publication bias was assessed by the visual inspection of funnel plots, formal testing by Egger’s regression asymmetry, and Begg’s rank correlation tests [31,32]; results were considered significant at *p* < 0.05.

We also conducted a dose–response meta-analysis to estimate the HRs for each 10% increment in UPF intake, according to the method introduced by Greenland and Orsini [33,34]. For this purpose, studies needed to report the number of cases and non-cases or person-years and median point of UPFs across more than three categories of UPF consumption. Ultimately, we conducted a one-stage linear mixed-effects meta-analysis to model the dose–response associations [35]. This method estimates the study-specific slope lines and combines them to obtain an average slope in a single stage. It includes studies with two categories of exposures in the dose–response analysis.

## 3. Results

### 3.1. Study Characteristics

**Figure 1:**
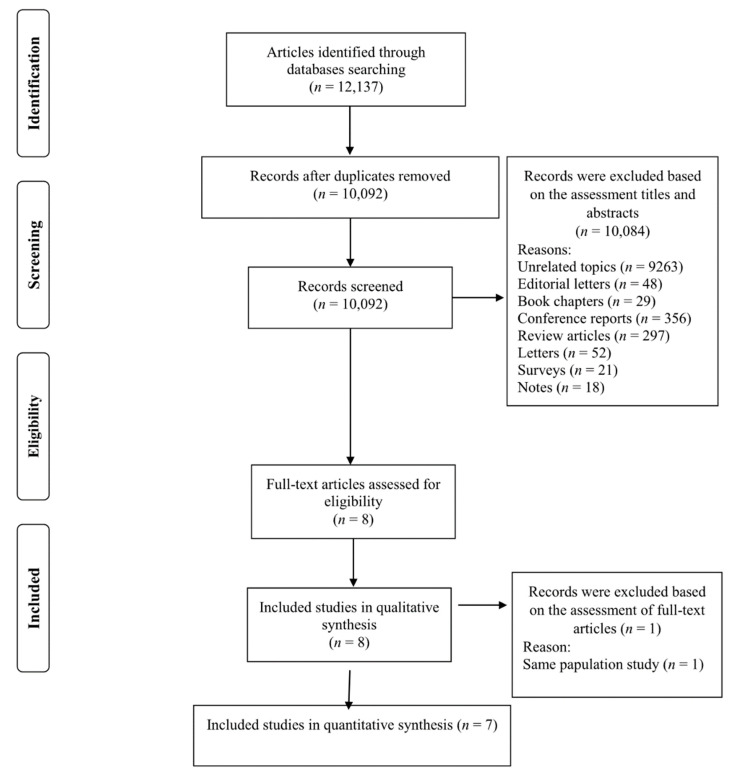
Flowchart of the process of the study selection.

We found 12,137 studies by a database search and reference lists. After removing duplicates, 10,092 records remained (Figure 1). The title and abstract of these studies were reviewed, and 10,084 studies were subsequently excluded on the basis of our inclusion criteria. Then, eight full-text studies were assessed, and one work was excluded because of the same population study [36] with another included study [37]. Finally, seven studies met our inclusion criteria and were included in the present work for the quantitative evaluation [17,19,20,21,22,37,38] (Figure 1).

The general characteristics of included studies are described in Table 1 and summarized below. All the included studies had a cohort design [17,19,20,21,22,37,38]. The study’s follow-up duration was between 2 and 27 years. These studies were published between 2019 and 2021 and were conducted in Spain [19,20,21], Italy [37], France [22], and USA [21,36].

The study-specific, maximally adjusted HR was reported for 207,291 participants across the selected work and was pooled for meta-analysis to evaluate the possible relationships between UPF consumption and mortality risk. Among these studies, the all-cause mortality risk was documented in four of them [19,20,37,38], four reported CVDs-cause mortality risk [17,19,37,38], two reported heart-cause mortality risk [17,37], and two reported the risk of cancer-cause mortality [19,36] as exposure factors. The included articles’ quality evaluation was completed applying the Newcastle–Ottawa scale [28], which indicated that all studies had high quality [17,19,20,21,22,37,38]. Moreover, our outcomes showed that the level of agreement between reviewers for data collection and quality evaluation was suitable (Kappa = 0.813).

### 3.2. Ultra-Processed Food Consumption and Mortality Risk

**Figure 2:**
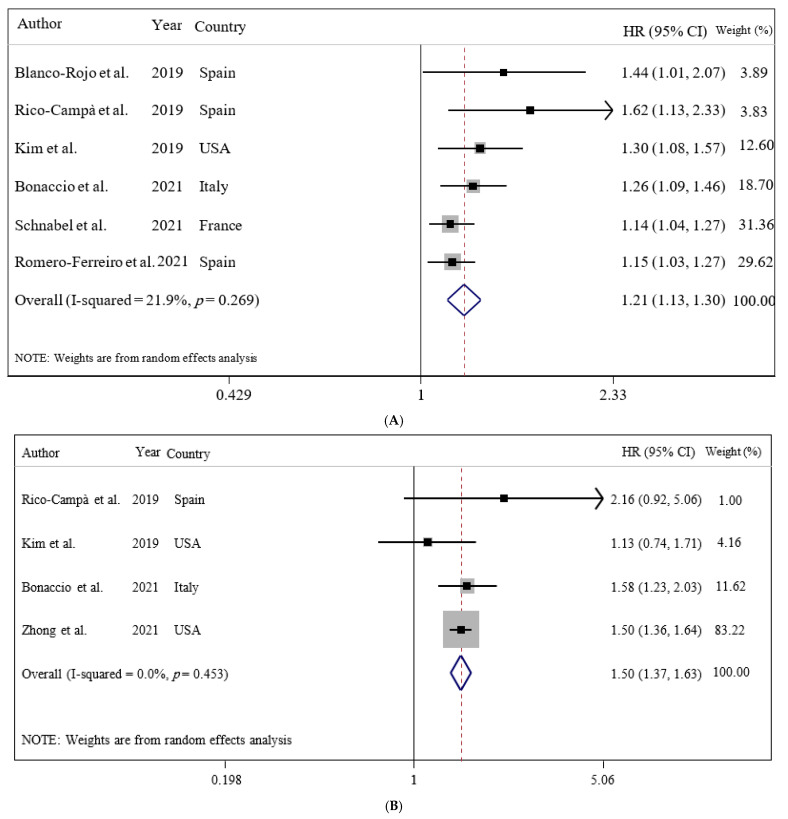

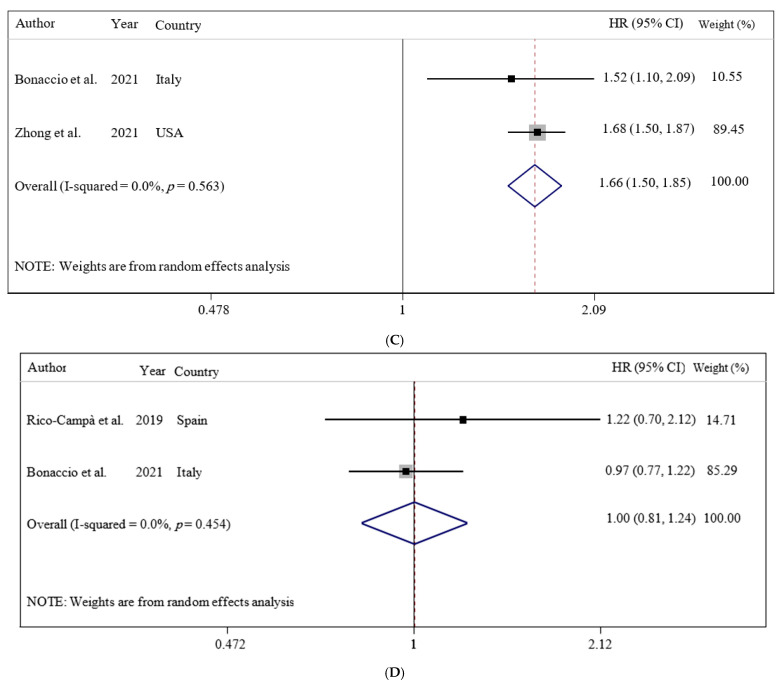
Forest plots demonstrating OR and 95% CI of pooled results from the random effects models to evaluate the relationship between ultra-processed food consumption and risk of mortality: (**A**) all-cause, (**B**) CVD-cause, (**C**) heart-cause, and (**D**) cancer-cause. The study-specific HR and 95 % CI are represented by black squares and horizontal lines, respectively; the area of the grey square is proportional to the specific-study weight to the overall meta-analysis. The center of the open diamond and the vertical dashed line represent the pooled HR, and its width represents the pooled 95 % CI.

Our results showed that UPF consumption was related to an enhanced risk of all-cause mortality (HR = 1.21; 95% CI: 1.13, 1.30; I^2^ = 21.9%; *p* < 0.001), CVDs-cause mortality (HR = 1.50; 95% CI: 1.37, 1.63; I^2^ = 0.0%; *p* < 0.001), and heart-cause mortality (HR = 1.66; 95% CI: 1.50, 1.85; I^2^ = 0.0%; *p* = 0.022), but not cancer-cause mortality (HR = 1.00; 95% CI: 0.81, 1.24; I^2^ = 0.0%; *p* = 0.976) (Figure 2). 

There were significant associations between UPF consumption and all-cause mortality risk among adults in all subgroups. However, subgroup analysis showed that UPF consumption was significantly associated with an enhanced risk of CVDs-cause mortality among adults with a body mass index (BMI) more than 25 (HR = 1.40; 95% CI: 1.02, 1.92; I^2^ = 44.8%; *p* = 0.039), but not less than 25 (Table 2).

**Figure 3:**
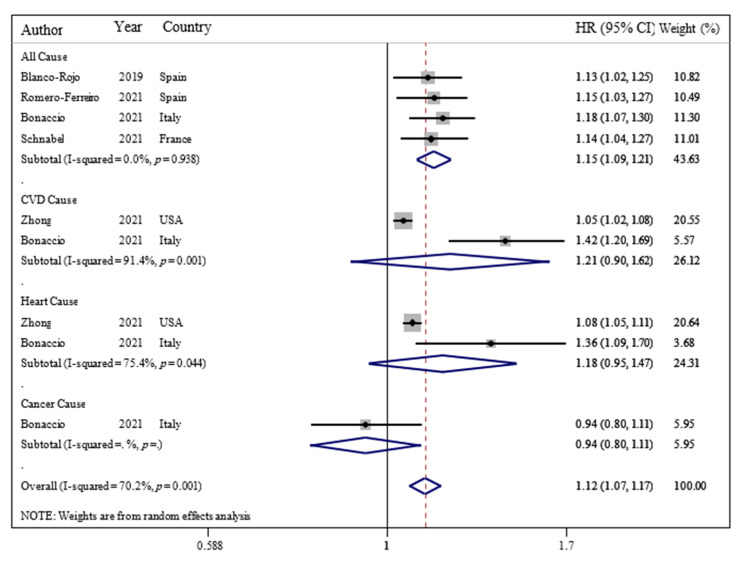
Forest plots showing the linear dose–response meta-analysis of mortality risk for 10% change in ultra-processed food consumption in daily intake. The study-specific HR and 95 % CI are represented by black squares and horizontal lines, respectively; the area of the grey square is proportional to the specific-study weight to the overall meta-analysis. The center of the open diamond and the vertical dashed line represent the pooled HR, and its width represents the pooled 95 % CI.

Furthermore, our findings revealed that each 10% increase in UPF consumption in daily calorie intake was associated with a 15% higher risk of all-cause mortality (OR = 1.15; 95% CI: 1.09, 1.21; I^2^ = 0.0%; *p* < 0.001) among adults (Figure 3).

**Figure 4:**
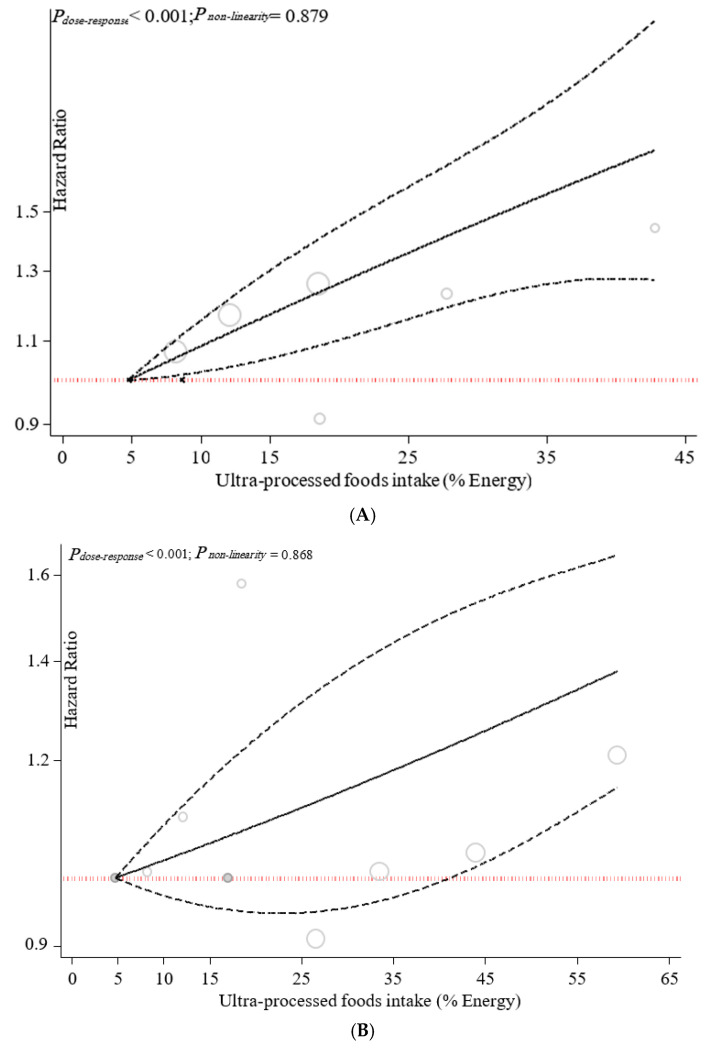

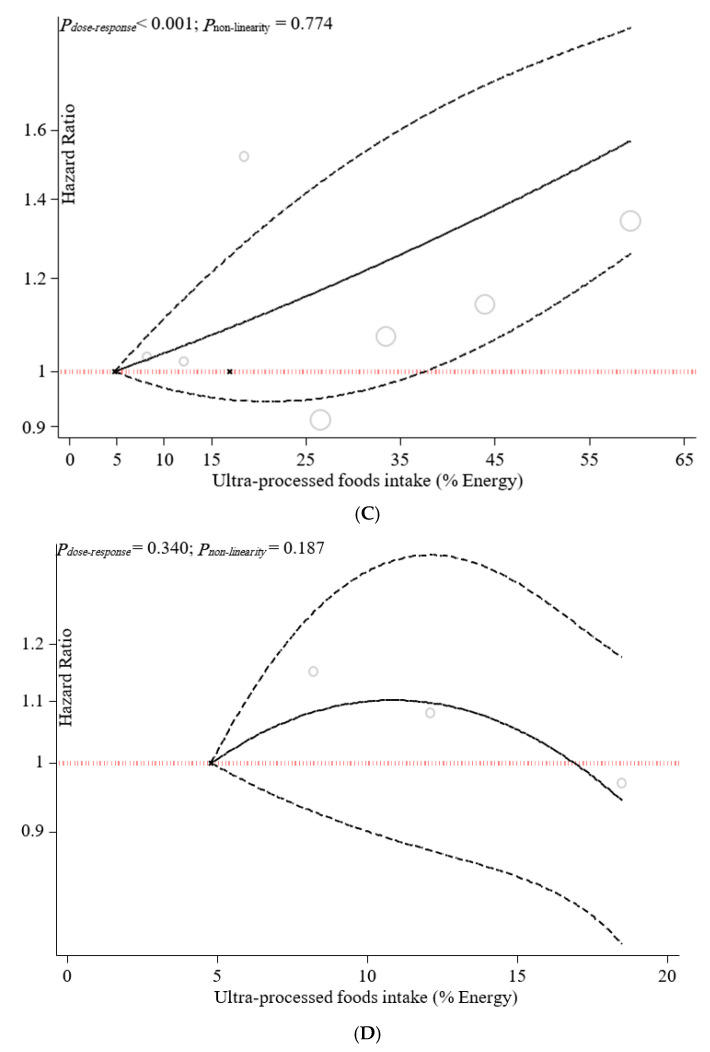
Dose–response association between ultra-processed food consumption and risk of mortality: (**A**) all-cause, (**B**) CVD-cause, (**C**) heart-cause, and (**D**) cancer-cause. It has been revised.

Dose–response associations are illustrated in Figure 4. The dose–response analysis revealed a positive linear association between UPF consumption and all-cause mortality (*p*_nonlinearity_ = 0.879, *p*_dose–response_ = *p* < 0.001), CVDs-cause mortality (*p*_nonlinearity_ = 0.868, *p*_dose–response_ = *p* < 0.001), and heart-cause mortality (*p*_nonlinearity_ = 0.774, *p*_dose–response_ = *p* < 0.001).

### 3.3. Sensitivity Analyses

**Figure 5:**
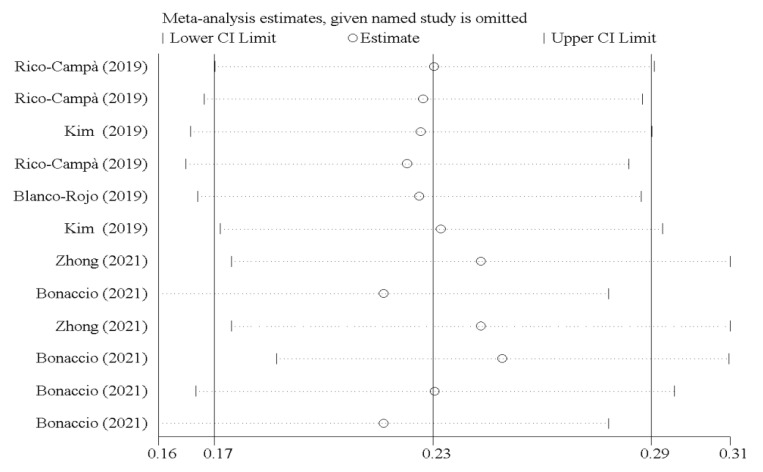
Forest plots showing sensitivity analysis results.

As illustrated in Figure 5, the study results were not affected by any study.

### 3.4. Publication Bias

**Figure 6:**
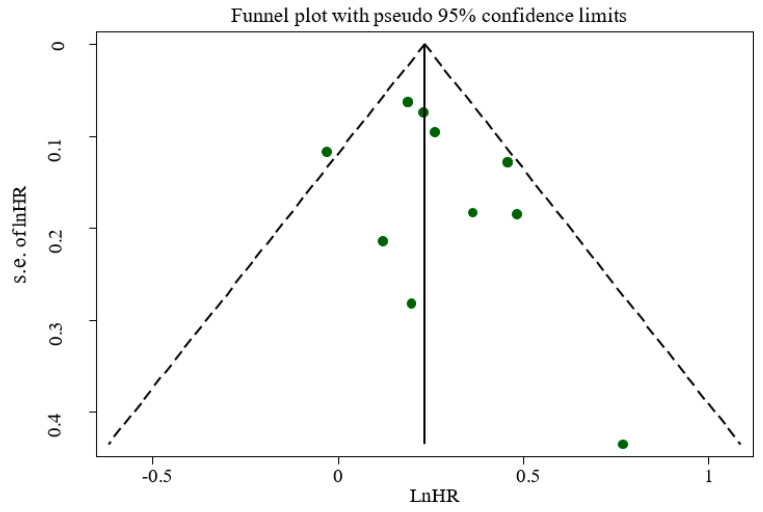
Funnel plot for evaluation publication bias.

The outcome of publication bias among studies did not show publication bias according to Egger’s regression asymmetry (*p* = 0.168) or Begg’s rank correlation tests (*p* = 0.217). This result was confirmed by a symmetric funnel plot (Figure 6).

## 4. Discussion

Globally, more than half of the deaths annually are due to CVDs and cancers [39]. A healthy diet plays a profound role in these conditions [40]. UPFs, food, and drink products that have undergone specified types of food processing have been shown to markedly increase the risk of mortality in many countries [19]. To our knowledge, the present systematic review and dose–response meta-analysis of seven cohort studies is the first investigation that evaluated the association between UPF consumption and risk of mortality in adults. According to the obtained results, UPF consumption was associated with an elevated risk of all-cause mortality, CVDs-cause mortality, and heart-cause mortality. However, there was no association between UPF consumption and cancer-cause mortality. Specifically, each 10% increase in UPF consumption in daily calorie intake was associated a 15% higher risk of all-cause mortality. In addition, the results of subgroup analysis proposed a significant positive association between UPF consumption and risk of CVDs-cause mortality among adults with a BMI of more than 25 kg·m^−2^. 

Similar to our results, a recent narrative review study by Matos et al. [41] concluded that consumption of UPFs is positively associated with the prevalence of chronic complications, including obesity, hypertension, CVDs, type 2 diabetes, and cancer, and consequently the risk of all-cause mortality. In addition, a prospective cohort study with 19,899 participants found that consumption of each additional serving of UPFs was associated with an 18% increase in all-cause mortality [19]. Furthermore, in a representative sample of USA adults, a higher frequency of UPF consumption was associated with a higher risk of all-cause mortality [38]. Additionally, Bonaccio et al. [36] reported that a high proportion of UPFs in the diet was associated with increased risk of CVDs and all-cause mortality. Furthermore, long-term results from a large prospective multicenter study demonstrated that high consumption of UPFs was related to increased risks of CVD mortality [17]. A recent prospective cohort by Ferriero et al. [21] indicated an inverse association between UPFs consumption and all-cause mortality. However, according to the conclusion of a systematic review performed by Marino et al. [11], since most of the observations about UPF consumption were derived from studies conducted with food questionnaires which are not explicitly validated for such foods, further efforts are essential to confirm the previously obtained results regarding consumption of UPFs and risk of mortality. 

Dietary patterns that involved a high content of vegetables, fruits, legumes, nuts, whole grains, unsaturated vegetable oils, and fish and a low content of red and processed meat, high-fat dairy, and refined carbohydrates were related to a decreased risk of mortality [42]. Emerging evidence suggests that consumption of UPF products characterized by low nutritional quality and high calorie content unfavorably contributes to an unhealthy dietary pattern, which elevates the risk of all-cause mortality as a substantial risk factor [41]. In addition, additives in such foods, including noncaloric artificial sweeteners, emulsifiers, and thickening agents such as carboxymethylcellulose (CMC) and carrageenan, cause various chronic disorders such as gut dysbiosis, metabolic dysfunction, systemic inflammation, endothelial dysfunction, and disrupted immune response [43,44,45]. As an additional layer of concern, synthetic compounds used in the packaging of UPFs, such as bisphenol A, can act as xenohormones. In particular, bisphenol A has been shown to impair reproductive function and increase the risk of cancer-cause mortality [18,46].

Despite several crucial strengths of the current quantitative review, including cohort design of all included studies, evaluating the association between UPFs consumption and risk of mortality for the first time, adjustment of findings for numerous probable confounders in the included studies, no evidence of publication bias, and performing a dose–response analysis, some potential limitations should be considered for interpreting our conclusions. Firstly, this investigation based on observational studies could not firm causation nor avoid the possibility of residual confounding for the proposed associations. Secondly, recall bias and misclassification of participants in terms of UPFs consumption were also possible. Thirdly, the component of UPFs varied across studies and could be dependent on the type of processing that food products have undergone. Lastly, dietary intake was assessed by 24 h dietary recall instead of food frequency questionnaire (FFQ) in two included studies.

The present dose–response meta-analysis showed that each 10% increase in UPF as a proportion of daily caloric intake was associated with a 15% higher risk of all-cause mortality. Although there was no association between UPF consumption and cancer-related mortality, a significant positive association was found between UPF consumption and cardiovascular disease-related mortality. Future longitudinal studies with sufficient control for confounding factors should focus on developing high-quality studies in diverse human populations to translate recommendations into practice. Several issues require further investigation in future studies. Existing instruments for assessing UPF intake are subjective and rather limited in scope, with most assessing only one aspect (i.e., cumulative UPF consumption). To more accurately assess the actual burden of UPF consumption, a specific food intake frequency questionnaire or dietary recording tool should be adapted or further developed to assess all aspects of UPF consumption, i.e., food class, specific components of UPF foods, their health effects, and specific procedures or additives. In addition, it is necessary to determine whether such associations are due to ultra-processing itself or to the nutritional or non-nutritional properties of UPF. Future studies should also investigate whether ultra-processing indices can demonstrate an association between diet and mortality compared with other nutritional quality scores/indices. Ultimately, assessment of associated variables such as genetic variants, lifestyle characteristics, demographic and socioeconomic status, and psychological disorders, as well as differences in therapy, may accelerate the discovery of potential mechanisms of UPFs in relation to mortality.

## Figures and Tables

**Table 1:** Characteristics of included studies.

Author (Year, Location)	Study Design/Follow-Up (Years)/Source of Data/Health Status	Population/Age/BMI/(Women/Men)	Ultra-Processed Food Assessment Method	Outcomes	Adjusted Variables	Quality Score
Blanco-Rojo et al. (2019, Spain)	Prospective cohort/7.7 years/the Study on Nutrition and Cardiovascular Risk in Spain (ENRICA)/healthy subjects	*N* = 11,898/age 55 ± 12 years/BMI = NR (6008/5890)	24 h recalls/NOVA food classification/frequency of ultra-processed food intake	Adults in the highest quartile versus the lowest of UPF consumption had higher risk of mortality (HR: 1.44; 95% CI: 1.01, 2.07).	Sex and age, educational level, living alone, smoking status, former drinker, physical activity index, time watching television, time devoted to other sedentary activities, the number of medications per day, and specific chronic conditions diagnosed by a physician	+8/10
Rico-Campà et al. (2019, Spain)	Prospective cohort/15 years/the Seguimiento Universidad de Navarra (SUN) project/healthy subjects	*N* = 19,899/age = 37.6 ± 12.3 years/BMI = 23.5 ± 3.5/(12,113/7786)	FFQ/NOVA food classification/frequency of ultra-processed food intake	UPF consumption had a higher hazard for all-cause mortality compared with those in the lowest quarter (HR = 1.62: 95% CI: 1.13 to 2.33).	Age, sex, marital status, physical activity, smoking status, snacking, special diet at baseline, body mass index, total energy intake, alcohol consumption, family history of cardiovascular disease, diabetes at baseline, hypertension at baseline, self-reported hypercholesterolemia at baseline, CVD at baseline, cancer at baseline, depression at baseline, education level and lifelong smoking stratified by recruitment period, deciles of age, sedentary index, and television viewing	+9/10
Kim et al. (2019, USA)	Prospective cohort/19 years/the Third National Health and Nutrition Examination Survey (NHANES III, 1988–1994)/healthy subjects	*N* = 11,898/age = 42 ± 0.5 years/BMI = 26.2 ± 0.2/(6067/5830)	FFQ/NOVA food classification/frequency of ultra-processed food intake	Higher frequency of ultra-processed food intake was associated with higher risk of all-cause mortality in a representative sample of US adults (HR = 1.31: 95% CI: 1.09 to 1.58).	Age, sex, race/ethnicity, total energy intake, poverty level, education level, smoking status, physical activity, alcohol intake, BMI, hypertension status, total cholesterol, and estimated glomerular filtration rate	+9/10
Bonaccio et al. (2021, Italy)	Prospective cohort/8.2 years/Moli-sani Study (2005–2010, Italy)/healthy subjects	*N* = 22,475/age = 55 ± 12/BMI = 28.2 ± 4.7/years/(10,702/11,733)	FFQ/NOVA food classification/proportion of UPF in the total weight of food and beverages consumed (g/day)	Adults in the highest quartile of UPF consumption had higher risk of CVD mortality (HR: 1.58; 95% CI: 1.23, 2.03).	Sex, age, energy intake, educational level, housing tenure, smoking, BMI, leisure time physical activity, history of cancer, CVDs, diabetes, hypertension, hyperlipidemia, and residence Mediterranean Diet Score	+9/10
Zhong et al. (2021, USA)	Prospective cohort/13.5 years/the Prostate, Lung, Colorectal, and Ovarian (PLCO)/healthy subjects	*N* = 91,891/age = >35 years/BMI = NR/(NR/NR)	FFQ/NOVA food classification/frequency of ultra-processed food intake	Participants in the highest vs. the lowest quintiles of ultra-processed food consumption had higher risks of death from cardiovascular disease (HR = 1.50; 95% CI: 1.36, 1.64) and heart disease (HR: 1.68; 95% CI: 1.50, 1.87) but not cerebrovascular disease (HR = 0.94; 95% CI: 0.76, 1.17).	Age, sex, race, educational, marital status, study center, aspirin use, history of hypertension, history of diabetes, smoking status, alcohol consumption, body mass index, physical activity, and energy intake from diet	+9/10
Schnabel et al. (2021, France)	Prospective cohort/2 years/the NutriNet-Santé Study/healthy subjects	*N* = 44 551/age = 56.7 ± 7.5 years/BMI = NR/(32,459/12,092)	24-h recalls/NOVA food classification/proportion of total energy	An increase in the proportion of UPF consumed was associated with a higher risk of all-cause mortality (HR = 1.14; 95% CI: 1.04, 1.27).	Sex, age, income level, education level, marital status, residence, BMI, physical activity level, smoking status, energy intake, alcohol intake, season of food records, first-degree family history of cancer or cardiovascular diseases, and number of food records	+8/10
Romero Ferreiro et al. (2021, Spain)	Prospective cohort/27 years/the multicenter study Diet and Risk of Cardiovascular Diseases (CVDs) in Spain (DRECE)/healthy subjects	*N* = 4679/age = 35.5 ± 15.6 years/BMI = 24.2 ± 5/(2391/2288)	FFQ/NOVA food classification/proportion of total energy	For every 10% of the energy intake from UPF consumption, an increase of 15% in the hazard of all-cause mortality was observed (HR, 1.15; 95% CI, 1.03–1.27).	Age, sex, BMI, physical activity, alcohol intake, smoking status and total energy intake, family history of CVDs, history of diabetes, hypertension, anger, myocardial infarction, and atherosclerosis	+9/10

UPF: ultra-processed foods, CVDs: cardiovascular diseases, HR: hazard ratio, FFQ: food frequency questionnaire, BMI: body mass index, NR: not reported.

**Table 2:** Subgroup analysis to assess the association between ultra-processed food consumption and the risk of mortality.

Subgrouped by	No. of Effect Size	HR ^1^	95% CI	*p*-Value	Heterogeneity		
					*p*-Values for within Groups	I^2^ (%)	*p*-Values for between Groups
**All-cause mortality**							
**Body mass index**							
Less than 25	1	1.62	1.13 to 2.33	0.009	<0.001	0.0	0.407
More than 25	2	1.28	1.14 to 1.43	<0.001	0.790	0.0	
**Assessment tools**							
Food record	1	1.44	1.01 to 2.07	0.046	<0.001	0.0	0.603
Food frequency	3	1.30	1.17 to 1.45	<0.001	0.454	0.0	
**Follow-up duration**							
Less than 10 years	2	1.28	1.12 to 1.47	<0.001	0.493	0.0	0.592
More than 10 years	2	1.37	1.14 to 1.65	0.001	0.295	10.8	
**Region**							
America	1	1.30	1.08 to 1.57	0.006	<0.001	0.0	0.888
Europe	3	1.32	1.16 to 1.50	<0.001	0.391	0.0	
**CVDs-cause mortality**							
**Body mass index**							
Less than 25	1	2.16	0.92 to 5.07	0.077	<0.001	0.0	0.175
More than 25	2	1.40	1.02 to 1.92	0.039	0.175	44.8	
**Follow-up duration**							
Less than 10 years	1	1.58	1.23 to 2.03	<0.001	<0.001	0.0	0.064
More than 10 years	3	1.22	1.08 to 1.37	0.001	0.397	0.0	
**Region**							
America	2	1.20	1.07 to 1.35	0.002	0.753	0.0	0.030
Europe	2	1.62	1.27 to 2.06	<0.001	0.496	0.0	

^1^ Calculated by random-effects model.

## Supplementary Materials

The following are available online at https://www.mdpi.com/article/10.3390/nu14010174/s1, Table S1: Search strategies including the key terms and the queries for each database, Table S2: Description of population, intervention, comparator and outcome (PICO).
