# Supplementary material for: Ultra-Processed Food Consumption and Adult Mortality Risk: A Systematic Review and Dose–Response Meta-Analysis of 207,291 Participants
Source: Nutrients. 2021 Dec 30;14(1):174. doi: 10.3390/nu14010174 (PMC8747520; doi:10.3390/nu14010174)
Supplement: Supplementary file 1 [file nutrients-14-00174-s001.zip › nutrients-1518511-supplementary.pdf]

Table S1. Search strategies including the key terms and the queries for each database.

**Database 8/8/2021**	**key terms and the queries**
PubMed (n=4827)	#1 ("fast foods"[All Fields] OR "fast foods"[MeSH Terms] OR "ultra processed food*"[All Fields] OR "ultraprocessed food*"[All Fields] OR "ultra processed food*"[All Fields] OR "processed food*"[All Fields] OR "ultra-processed"[All Fields] OR "ultraprocessed"[All Fields] OR "ultra-processed"[All Fields] OR "NOVA"[All Fields] OR "nova food classif*"[All Fields] OR "nova food*"[All Fields] OR "nova food classif*"[All Fields] OR "NOVA food classification system"[All Fields])<br><br>#2 ("Mortality"[MeSH Terms] OR "Mortality"[Title/Abstract] OR "Death"[Title/Abstract] OR "Fatal"[Title/Abstract] OR "survive"[Title/Abstract] OR "survival"[Title/Abstract])<br><br>#3 #1 AND #2
Web of Science (n=1763)	#1 TOPIC: ("fast foods") OR TOPIC: ("ultra processed food*") OR TOPIC: ("ultraprocessed food*") OR TOPIC: ("ultra processed food*") OR TOPIC: ("processed food*") OR TOPIC: ("ultra-processed") OR TOPIC: ("ultraprocessed") OR TOPIC: ("ultra-processed") OR TOPIC: ("NOVA") OR TOPIC: ("nova food classif*") OR TOPIC: ("nova food*") OR TOPIC: ("nova food classif*") OR TOPIC: ("NOVA food classification system")<br><br>#2 TOPIC: ("Mortality") OR TOPIC: ("death") OR TOPIC: ("Fatal") OR TOPIC: ("survive") OR TOPIC: ("survival")<br><br>#3 #1 AND #2
Scopus (n=5547)	#1 (TITLE-ABS-KEY ("ultra-processed food*") OR TITLE-ABS-KEY ("ultraprocessed food*") OR TITLE-ABS-KEY ("ultra processed food*") OR TITLE-ABS-KEY ("processed food*") OR TITLE-ABS-KEY ("ultra processed") OR TITLE-ABS-KEY ("ultraprocessed") OR TITLE-ABS-KEY ("ultra-processed") OR TITLE-ABS-KEY ("NOVA") OR TITLE-ABS-KEY ("NOVA food classif*") OR TITLE-ABS-KEY ("NOVA food*") OR TITLE-ABS-KEY ("NOVA food classif*") OR TITLE-ABS-KEY ("NOVA food classification system") OR TITLE-ABS-KEY ("fast foods"))<br><br>#2 ((TITLE-ABS-KEY ("Mortality") OR TITLE-ABS-KEY ("Death") OR TITLE-ABS-KEY ("Fatal") OR TITLE-ABS-KEY ("survive") OR TITLE-ABS-KEY ("survival")))<br><br>#3 #1 AND #2

Table S2. Description of population, intervention, comparator and outcome (PICO).

**Population**	Adults (≥18 years)
**Intervention**	-
**Comparison**	Ultra-processed Food Consumption
**Outcome**	Mortality Risk
